# Delaying the oocyte maturation trigger by one day leads to a higher metaphase II oocyte yield in IVF/ICSI: a randomised controlled trial

**DOI:** 10.1186/1477-7827-12-31

**Published:** 2014-04-23

**Authors:** Frank Vandekerckhove, Jan Gerris, Stijn Vansteelandt, An De Baerdemaeker, Kelly Tilleman, Petra De Sutter

**Affiliations:** 1 Centre for Reproductive Medicine, University Hospital Ghent, De Pintelaan 185, Gent 9000, Belgium; 2 Department of Applied Mathematics, Computer Science and Statistics, Ghent University, Krijgslaan 281 S9, Gent 9000, Belgium

## Abstract

**Background:**

The negative impact of rising progesterone levels on pregnancy rates is well known, but data on mature oocyte yield are conflicting. We examined whether delaying the oocyte maturation trigger in IVF/ICSI affected the number of mature oocytes and investigated the potential influence of serum progesterone levels in this process.

**Methods:**

Between January 31, 2011, and December 31, 2011, 262 consecutive patients were monitored using ultrasound plus hormonal evaluation. Those with > =3 follicles with a mean diameter of > =18 mm were divided into 2 groups depending on their serum progesterone levels. In cases with a progesterone level < = 1 ng/ml, which was observed in 59 patients, 30-50% of their total number of follicles (only counting those larger than 10 mm) were at least 18 mm in diameter. These patients were randomised into 2 groups: in one group, final oocyte maturation was triggered the same day; for the other, maturation was triggered 24 hours later. Seventy-two patients with progesterone levels > 1 ng/ml were randomised in the same manner, irrespective of the percentage of larger follicles (> = 18 mm). The number of metaphase II oocytes was our primary outcome variable. Because some patients were included more than once, correction for duplicate patients was performed.

**Results:**

In the study arm with low progesterone (<= 1 ng/ml), the mean number of metaphase II oocytes (+/-SD) was 10.29 (+/-6.35) in the group with delayed administration of the oocyte maturation trigger versus 7.64 (+/-3.26) in the control group. After adjusting for age, the mean difference was 2.41 (95% CI: 0.22-4.61; p = 0.031). In the study arm with elevated progesterone (>1 ng/ml), the mean numbers of metaphase II oocytes (+/-SD) were 11.81 (+/-9.91) and 12.03 (+/-7.09) for the delayed and control groups, respectively. After adjusting for PCOS (polycystic ovary syndrome) and female pathology, the mean difference was -0.44 (95% CI: -3.65-2.78; p = 0.79).

**Conclusions:**

Delaying oocyte maturation in patients with low progesterone levels yields greater numbers of mature oocytes.

**Trial registration:**

B67020108975 (Belgian registration) and NCT01980563 (ClinicalTrials.gov).

## Background

Various ultrasound and hormonal criteria have been used to determine the moment to trigger oocyte maturation in IVF/ICSI cycles. Historically, the moment for triggering oocyte maturation has been based on follicle diameters that were measured using ultrasound and levels of serum estradiol [1,2]. A Cochrane review [3] stated that the use of sonographic criteria alone might be sufficient but that the simultaneous determination of serum estradiol is still recommended as long as large randomised controlled trials have not shown that the incidence of ovarian hyperstimulation syndrome is equal in both groups. Combined monitoring was recommended as "a precautionary good practice point". These findings were mainly gathered in agonist protocols.

The decision to advance trigger oocyte maturation by 24 hours did not seem to have a positive effect on the probability of pregnancy in an antagonist protocol [4].

Irrespective of the use of an agonist or an antagonist for suppression, we tested whether the protocol of Garcia-Velasco et al. [5], in which oocyte maturation is triggered as soon as 3 follicles reach diameters of 18 mm, could be adapted further. These authors used it to compare ovarian steroid production when either an agonist or an antagonist was used. We also applied a uniform protocol for monitoring and hypothesised that delaying the administration of the trigger for 24 hours would result in a higher yield of mature oocytes, which served as our primary outcome variable. To evaluate whether this modification had any consequences on pregnancy rates or pregnancy outcomes, these variables were further evaluated as secondary outcomes. This randomised controlled trial was performed in patients with normal serum progesterone levels.

It is known that high progesterone levels (>1.5 ng/ml) have a deleterious effect on the endometrium and, thus, on pregnancy rates [6]. When progesterone levels are slightly elevated (>1 ng/ml), it can be difficult to decide whether to continue the stimulation procedure for one more day. A randomised controlled trial was carried out on this group of patients to evaluate the number of mature oocytes at retrieval as the primary outcome variable. Pregnancy rates and outcomes were important secondary variables.

## Methods

This study was approved by the Ethical Committee of the Ghent University Hospital (B67020108975) and as a clinical trial internationally (NCT01980563 at ClinicalTrials.gov). It was part of a larger prospective trial in a single university hospital (Gent, Belgium) that compared cycle monitoring for IVF/ICSI in two parallel control groups: those with ultrasound monitoring versus those with combined monitoring (ultrasound plus hormonal monitoring). Between January 31, 2011, and December 31, 2011, 262 consecutive patients from the latter group were eligible for the present trial.

Inclusion criteria were the presence of both ovaries and being a female less than 45 years of age. Patients with ovarian cysts at the start of the ovarian stimulation procedure were excluded. Ovarian reserve was determined by measuring anti-Müllerian hormone (AMH) before starting treatment (Immunotech, Beckman Coulter Company, Brea, CA, USA).

Various protocols for controlled ovarian hyperstimulation were applied. Either recombinant FSH (Gonal F®, Merck Serono, Geneva, Switzerland) or urinary FSH (Menopur®, Ferring Pharmaceuticals, Saint-Prex, Switzerland) was used with daily doses between 150 and 300 U, dependent on age, anti-Müllerian hormone (AMH) levels and previous response, if applicable. In the agonist group, 0.1 mg triptorelin (Decapeptyl®, Ipsen, Paris, France) was administered subcutaneously for 7 days starting on cycle day 1, and gonadotrophins were started on cycle day 3. In the antagonist group, a fixed protocol was used: gonadotrophins were started on cycle day 3, and 0.25 mg cetrorelix (Cetrotide®, Merck Serono, Geneva, Switzerland) was injected subcutaneously as a daily dose from the 6th day of stimulation until the day of oocyte maturation triggering. After 1 week of stimulation with gonadotrophins, a first ultrasound monitoring session was planned. Serum levels of estradiol, LH and progesterone were determined simultaneously. All samples were analysed with ECLIA (Modular E170, Roche, Vilvoorde, Belgium). The inter- and intra-assay coefficients of variability for the progesterone assay were 3.46–6.71% and 1.1–7.0%, respectively. The cut-off for the sensitivity of the test (minimal detectable level) was 0.15 ng/ml. Depending on the findings, patients were scheduled for additional monitoring every 1 or 2 days. As soon as three follicles reached a diameter of at least 18 mm, patients were divided into two groups: those with serum progesterone > 1 ng/ml and those with a low progesterone level, defined as ≤ 1 ng/ml. The results of the individual monitoring of the patients were centralised and discussed at a daily staff meeting. All 6 staff members who were performing monitoring enrolled patients equally.

When the serum progesterone level was > 1 ng/ml, patients were randomised (single blinded). They received 5000 U of human chorionic gonadotrophin (hCG) (Pregnyl®, Merck Sharp & Dohme, NJ, USA) on the same day (high progesterone early [HPE] group) or 24 hours later (high progesterone late [HPL] group). A computer-generated list was used, and allocation concealment was performed by a secretary over the telephone. This procedure was supervised by a single observer (FV). No further monitoring was planned the day after randomisation into the HPL group. When more than 3 oocytes were expected, oocytes were retrieved with a single lumen needle. Flushing through a double lumen needle was performed in all other cases. All laboratory procedures were carried out as previously described [7]. In cases of ICSI treatment, the number of mature oocytes was dependent on their morphological appearance after denudation. In IVF cycles, all oocytes that were inseminated were classified as mature, as is acceptable convention per the literature. A maximum of 2 embryos were transferred 3 days after oocyte retrieval. Luteal support consisted of 600 mg micronised progesterone (Utrogestan®, Besins Healthcare, Bangkok, Thailand) that was administered vaginally in three daily doses, starting after oocyte collection, and continued until 2 weeks after transfer if not pregnant or until a clinical pregnancy was confirmed by ultrasound.

For patients with a low progesterone level (<1 ng/ml), a different approach was used. When > 50% of the follicles were larger than 18 mm, 5000 U of hCG was injected on the same day. Only follicles of at least 10 mm were counted to obtain this ratio. If the number of follicles with a diameter of at least 18 mm was between 30 and 50% of the total number counted, the patient was randomised. They received 5000 U of hCG on the same day (low progesterone early [LPE] group) or 24 hours later (low progesterone late [LPL] group), and no extra monitoring procedure was performed the day after randomisation in the LPL group. Allocation was performed as previously described. Further treatment was completed as described above for patients with a progesterone level > 1 ng/ml.

The results of the trial were processed anonymously by a single observer (FV). The number of metaphase II oocytes (MII) was our primary outcome variable. Secondary variables that demonstrate the oocyte yield were the number of oocytes retrieved, the number of fertilised oocytes (2PN) and the number of good quality embryos (GQE) on day 3. Other secondary outcome variables were defined according to the literature [8]: pregnancy rates (PR), clinical pregnancy rates (CPR), ongoing pregnancy rates (OPR) and live birth rates (LBR) as expressed per cycle; clinical implantation ratios (CIR/E), ongoing implantation ratios (OIR/E) and live birth ratios (LBR/E) were calculated for each individual embryo that was transferred.

The sample size calculation for the group with progesterone levels ≤ 1 ng/ml was based on a mean yield of 6 mature oocytes (SD = 3) in the LPE group versus 11 (SD = 6) in the LPL group [4,9], resulting in a required sample size of 15 in each group (Welch’s t-test, 5% significance level, 80% power). For patients with high progesterone levels (>1 ng/ml), we found no comparable data in the literature as a reference. We therefore decided to include patients in the HPE and HPL groups concomitantly with the LPE and LPL groups.

The descriptive analyses in Table 1 were based on Fisher's exact tests for proportions and Student’s t-tests for continuous outcomes; when the data were skewed or contained outliers, the non-parametric Mann–Whitney U-test was used. To account for correlations between measurements for women with repeated cycles, all further analyses were based on linear and logistic marginal regression models that were fitted using generalised estimating equations with exchangeable working correlations. Although adjustment for baseline covariates was not required in view of the randomised study design, adjustments for age, PCOS and female pathology were used to improve precision in some of the linear models. All tests were performed at the 5% significance level. Statistical analyses were performed using SPSS, version 21, and R Studio, version 0.97.320.

**Table 1:** Descriptive statistics

**Variable**	**Prog ≤ 1 ng/ml**	**Prog ≤ 1 ng/ml**	**Prog > 1 ng/ml**	**Prog > 1 ng/ml**
	**hCG day 0 (LPE)**	**hCG day +1 (LPL)**	**hCG day 0 (HPE)**	**hCG day +1 (HPL)**
*Number of patients [cycles]*	20 [28]	31 [31]	28 [36]	25 [36]
*Age (years)*	34.5 (4.4)	33.5 (4.9)	30.5 (3.8)	31.9 (4.2)
*Gravidity*	0.9 (1.3)	0.9 (1.7)	0.5 (0.9)	0.8 (1.4)
*Parity*	0.4 (0.6)	0.4 (0.8)	0.3 (0.6)	0.3 (0.9)
*Duration of infertility (years)*	3.3 (2.1)	4.3 (1.4)	4.3 (1.9)	5.2 (2.9)
AMH (ng/ml)*	2.7 (2.1 - 5.0)	4.1 (2.5 - 6.2)	4.6 (3.2 - 5.7)	3.0 (2.3 - 5.2)
E2 (pg/ml)*	1460 (956–2150)	1390 (964–2110)	1298 (810–1825)	1130 (866–1978)
Progesteron (ng/ml)*	0.7 (0.5 - 0.9)	0.8 (0.5 - 0.9)	1.5 (1.2 - 1.8)	1.4 (1.2 - 1.6)
*Number of embryos transferred*	1.25 (0.64)	1.52 (0.72)	1.29 (0.60)	1.28 (0.46)
*Female pathology*	6/20	10/31	16/28	9/25
*Endometriosis*	4/20	2/31	7/28	0/25
*Ovarian dysfunction*	0/20	2/31	3/28	1/25
*PCOS*	0/20	1/31	0/28	2/25
*Male pathology*	7/20	12/31	13/28	14/25
*Antagonist*	17/28	15/31	17/36	19/36
*Recombinant FSH*	20/28	24/31	28/36	34/36
*Experienced performer transfer*	20/28	19/31	22/36	22/36

Numbers are expressed as numbers or as means (SD) unless explained differently.

*Numbers are expressed as median (first and third quartile).

p > 0.05 for all variables.

Subsequent to ending the trial, additional evidence came to suggest that serum progesterone levels > 1.5 ng/ml at the moment of triggering oocyte maturation might lower pregnancy rates. Therefore, we performed an additional comparison of 2 subgroups of patients: those with low progesterone levels (<1.0 ng/ml; group A) and those with highly elevated levels (>1.5 ng/ml; group B). Fisher's exact test and Student's t-test were again used as described above.

## Results

**Figure 1:**
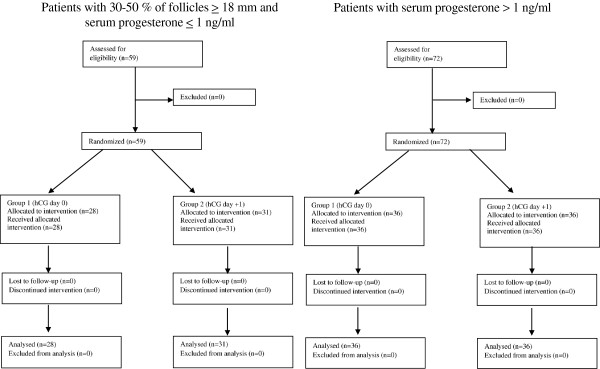
CONSORT flow chart.

Seventy-two patients with at least three follicles ≥ 18 mm had serum progesterone levels > 1 ng/ml. They were randomised into 2 groups (HPE and HPL) of 36 individuals each. In the HPE group, the oocyte maturation trigger was administered on the same day. In the HPL group, hCG was injected 24 hours later. In the remaining cases with low serum progesterone levels (<1 ng/ml), 59 patients had 30 to 50% of their follicles measuring equal to or greater than 18 mm. After randomisation, 28 patients were allocated to the LPE group and received hCG on the same day. The remaining 31 patients (LPL group) were triggered 24 hours later. All eligible patients were randomised and could be allocated (Figure 1). The data were analysed after correcting for duplicate patients in each group.

No important differences were observed between the LPE and LPL groups and between the HPE and HPL groups for all controlled variables (Table 1): age of the female, gravidity, parity, duration of infertility, ovarian reserve determined by AMH, peak estradiol level, number of transferred embryos, diagnostic criteria (such as female pathology, endometriosis, ovarian dysfunction and PCOS), male pathology, stimulation protocol, number of cancellations, number of failed fertilisations and the experience of the physician performing the embryo transfer.

### What is the effect of delaying the oocyte maturation trigger by 24 hours in patients with low serum progesterone (<1 ng/ml)?

**Figure 2:**
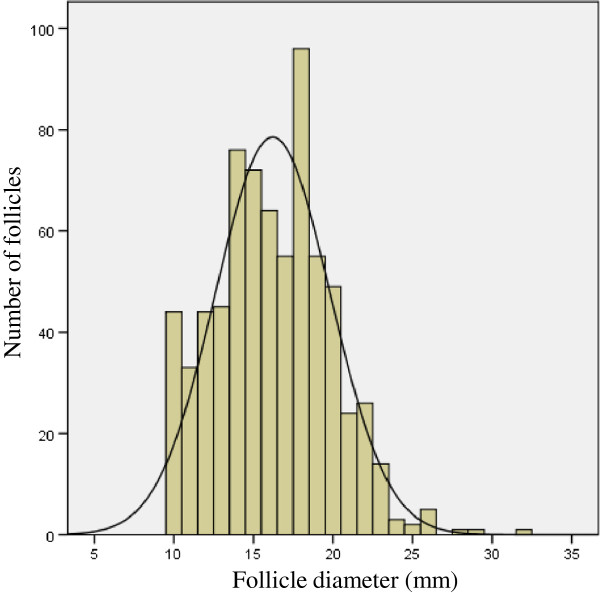
The distribution of follicle diameters in patients with normal serum progesterone (<1 ng/ml).

In Figure 2, the distribution of the follicle diameters in these patients is illustrated. By waiting 24 hours (LPL group), we obtained more oocytes and predominantly more mature ones compared with the LPE group, where hCG was administered on the same day (Table 2). Multivariate analysis with correction for the female’s age showed a significant difference in the number of oocytes (p = 0.021) and in the number of mature oocytes (p = 0.031) in favour of the LPL group (Table 2). No statistically significant differences were found in the number of fertilised oocytes and the number of good-quality embryos.

**Table 2:** The influence of delaying oocyte maturation in patients with a normal progesterone level on the yield of (mature) oocytes, fertilised oocytes and good quality embryos

**Day hCG**	**Number included (cycles/[patients])**	**Oocytes**	**MII**	**2PN**	**GQE**
0 (LPE)	28 [20]	9.36 (3.21)	7.64 (3.26)	5.29 (3.26)	4.32 (2.62)
+1 (LPL)	31 [31]	12.58 (7.62)	10.29 (6.35)	7.48 (5.25)	5.74 (4.61)
	**Mean difference**	2.97	2.41	1.80	1.18
	95% CI	0.45 to 5.49	0.22 to 4.61	-0.15 to 3.76	-0.53 to 2.88
	P	0.021	0.031	0.071	0.18

Numbers are expressed as means (SD).

Adjusted for age.

Several secondary variables were evaluated to compare the pregnancy rates and pregnancy outcomes. No significant differences were found between groups LPE and LPL (Table 3). Multivariate analysis did not influence the final results.

**Table 3:** The influence of delaying oocyte maturation in patients with a normal progesterone level on pregnancy and pregnancy outcome

**Day hCG**	**PR/cycle**	**CPR/cycle**	**OPR/cycle**	**LBR/cycle**		CIR/E*	OIR/E*	LBR/E*
*0 (LPE)*	31.3%	31.3%	27.1%	27.1%		0.27 (0.42)	0.22 (0.40)	0.22 (0.40)
*+1 (LPL)*	35.5%	29.0%	25.8%	25.8%		0.26 (0.43)	0.24 (0.43)	0.24 (0.43)
**Odds ratio**	1.24	0.92	1.00	1.00	**Mean difference**	-0.017	0.017	0.017
95% CI	0.40 to 3.86	0.29 to 2.92	0.30 to 3.37	0.30 to 3.37	95% CI	-0.23 to 0.20	-0.20 to 0.23	-0.20 to 0.23
P	0.71	0.88	1.00	1.00	P	0.88	0.87	0.87

*Numbers are expressed as means (SD).

Unadjusted.

### What is the effect of delaying the oocyte maturation trigger by 24 hours in patients with high serum progesterone (>1 ng/ml)?

**Figure 3:**
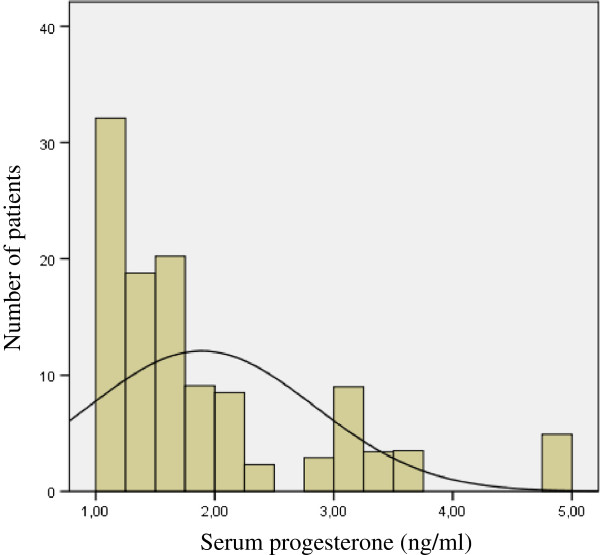
The distribution of progesterone levels in patients with elevated serum progesterone (>1 ng/ml).

Figure 3 reveals a non-normal distribution of elevated progesterone levels. Thirty patients (48 cycles) had moderately elevated serum progesterone levels (>1 ng/ml and ≤ 1.5 ng/ml). Levels higher than 1.5 ng/ml were found in 23 patients (24 cycles). Cases with very high progesterone levels (>3 ng/ml) were very scarce (3 patients).

The yield of mature oocytes was not different between the HPE (hCG administered the same day) and HPL (hCG administered 24 hours later) groups. Multivariate analysis did not affect the results (Table 4). The other variables (viz., number of oocytes recovered, fertilised oocytes, good quality embryos and all variables describing implantation and pregnancy rate) were also comparable (Table 5).

**Table 4:** The influence of delaying oocyte maturation in patients with an elevated progesterone level on the yield of (mature) oocytes, fertilised oocytes and good quality embryos

**Day hCG**	**Number included (cycles/[patients])**	**Oocytes**	**MII**	**2PN**	**GQE**
0 (HPE)	36 [28]	14.23 (8.44)	12.03 (7.09)	8.22 (5.84)	6.5 (5.02)
+1 (HPL)	36 [25]	13.06 (10.00)	11.81 (9.91)	7.78 (5.86)	6.2 (4.80)
	**Mean difference**	-1.52	-0.44	-0.97	-0.75
	95% CI	-5.30 to 2.27	-3.65 to 2.78	-3.49 to 1.55	-2.98 to 1.48
	P	0.43	0.79	0.45	0.51

Numbers are expressed as numbers or as mean (SD).

Adjusted for PCO and female pathology.

**Table 5:** The influence of delaying oocyte maturation in patients with an elevated progesterone level on pregnancy and pregnancy outcome

**Day hCG**	**PR/cycle**	**CPR/cycle**	**OPR/cycle**	**LBR/cycle**		CIR/E*	OIR/E*	LBR/E*
*0 (HPE)*	20.7%	20.1%	17.6%	17.6%		0.16 (0.33)	0.15 (0.33)	0.15 (0.33)
*+1 (HPL)*	31.8%	28.8%	23.2%	23.2%		0.23 (0.38)	0.18 (0.36)	0.18 (0.36)
**Odds ratio**	1.75	1.61	1.41	1.41	**Mean difference**	0.063	0.033	0.033
95% CI	0.59 to 5.22	0.54 to 4.78	0.46 to 4.34	0.46 to 4.34	95% CI	-0.10 to 0.22	-0.12 to 0.18	-0.12 to 0.18
P	0.31	0.39	0.55	0.55	P	0.44	0.67	0.67

*Numbers are expressed as means (SD).

Unadjusted.

### What is the effect of highly elevated serum progesterone (>1.5 ng/ml)?

We also compared the findings between patients with low progesterone levels (group A) and patients with very high progesterone levels (>1.5 ng/ml) (group B) who were triggered on the day of the final monitoring (i.e., no delay of 24 hours). The numbers of (mature) oocytes, fertilised oocytes and good quality embryos were all significantly higher in group B. The variables describing implantation and pregnancy outcome were lower in group B, although statistical significance was lacking. More data are required to confirm our findings (Table 6).

**Table 6:** The oocyte yield and pregnancy outcome in subgroups categorized by highly different progesterone levels

	**Group A**	**Group B**	**P**
	**Prog ≤ 1 ng/ml D0**	**Prog > 1,5 ng/ml D0**	
**Number of patients included**	28	14	
**Number of oocytes**	9,36 (3,21)*	16,71 (9,93)*	0.02
**Number of mature oocytes**	7,64 (3,26)*	13,43 (8,36)*	0.03
**Number of fertilised oocytes**	5,29 (3,26)*	10,00 (6,50)*	0.02
**Number of good quality embryos**	4,32 (2,62)*	7,93 (5,14)*	0.03
**PR/cycle**	31.3%	7,14%	0.07
**CPR/cycle**	31.3%	7,14%	0.07
**OPR/cycle**	27.1%	7,14%	0.11
**LBR/cycle**	27.1%	7,14%	0.11
**CIR/embryo**	0.27 (0.42)	0.07 (0.27)	0.07
**OIR/embryo**	0.22 (0.40)	0.07 (0.27)	0.16
**LBR/embryo**	0.22 (0.40)	0.07 (0.27)	0.16

Numbers are expressed as numbers or as means (SD) unless explained differently.

*p < 0.05.

## Discussion

### The effect of increasing progesterone levels

Increasing serum progesterone during stimulation for IVF/ICSI may have a negative impact on pregnancy rates. Venetis [10] reviewed this phenomenon and included studies with progesterone levels ≥ 0.9 ng/ml. Initially, this effect was described in only a fraction of the reports. In a recent systemic review [11], he expanded and further confirmed his findings. It seemed that a ratio of progesterone-to-estradiol > 0.48 reduced pregnancy rates in antagonist cycles and that this ratio was found to be an independent predictor of pregnancy [12]. The effect of elevated progesterone levels was higher in patients with a rather low ovarian response [11]. An emerging progesterone increase could be predicted by the number of follicles and an increase in serum estradiol [13]. This possible negative effect was the consequence of endometrial changes, because it was not described in oocyte donation programs [14] or when embryos that were obtained in a cycle with high progesterone were cryopreserved and subsequently thawed and transferred [15]. Van Vaerenbergh [16] demonstrated that gene expression in the endometrium thoroughly changed when serum progesterone levels were higher than 1.5 ng/ml. This threshold of 1.5 ng/ml was further used by Bosch [6], who clearly demonstrated a negative effect on pregnancy rates in both agonist and antagonist cycles. Although our study was not powered to compare implantation and pregnancy rates, the additional data in Table 6 confirmed these findings.

The yield of mature oocytes was either not mentioned or only calculated as a secondary variable in most studies. Mio [17], Bustillo [18] and Venetis [11] demonstrated a higher number of retrieved oocytes in cycles with elevated progesterone levels. The cut off values for positive progesterone levels differed between studies, and uniform conclusions were not formulated. In our own study (Table 4), there was no significant difference in the number of (mature) oocytes.

From these observations, we may conclude that there is no evidence that increasing progesterone levels have a negative effect in cases where eggs are recruited for donation or for further cryopreservation, be it for medical or non-medical reasons.

Another possibility is that, if rising progesterone levels are encountered during the stimulation procedure, we can delay for a few days more to yield a maximum number of good-quality eggs. They can be fertilised and cryopreserved and be used for transfer later on, the so-called “segmented procedure” [19].

### The importance of follicle diameters

The ultrasound criteria to decide the best moment for triggering oocyte maturation have always been a point of discussion.

When no GnRH agonist/antagonist has been used, a leading follicle diameter of 16 mm or more and a serum estradiol of at least 600 pg/ml have served as guidelines for administering 10000 IU hCG [20]. Using a GnRH agonist—either in a long or short protocol—multiple criteria have been suggested. This can be explained by the different stimulation protocols that have been used and the variations in study designs. The leading follicles have had to reach diameters of 16 to 20 mm in most cases [1,2,21-28].

In antagonist cycles, most studies have proposed leading follicle diameters of 16 to 17 mm [4,9,29-31]. It seems that, in cycles where an antagonist has been used, the decision has been made somewhat earlier than in agonist cycles. In 2006, the Brussels GnRH antagonist Consensus Workshop Group stated that the optimal timing for triggering oocyte maturation when using a GnRH antagonist protocol needed to be explored further [32].

In our study, we focused on follicle diameters in selected patients with low progesterone levels (<1 ng/ml). They all reached the threshold of having a least 3 follicles ≥ 18 mm [5] with 30–50% of them being large enough. When waiting 24 hours to inject hCG, a larger number of (mature) oocytes were obtained (Table 2), as has already been mentioned by others [27].

In our series, we could not confirm a higher pregnancy rate in the group in which we triggered oocyte maturation 1 day later (Table 3). This confirms the findings by Tremmelen and Lane [33], who found that advancing or delaying hCG administration by 1 day from ‘ideal’ had no adverse impact on IVF treatment outcomes in non-programmed GnRH antagonist cycles. Again, we must notice that our study was not powered to compare pregnancy rates, so conclusions on cycle outcomes must be interpreted with caution.

In spite of this, our findings support the idea that a higher yield of mature oocytes indirectly contributes to a higher overall productivity rate, as mentioned by Stanger and Yovich [34].

## Conclusions

As soon as three follicles have a diameter ≥ 18 mm, further decisions to pinpoint the moment for administering hCG depend on the progesterone level. If the progesterone level is higher than 1 ng/ml, delaying the administration of hCG by 24 hours has no effect on the number of mature oocytes. If the progesterone level is ≤ 1 ng/ml and 30–50% of the follicles have diameters ≥ 18 mm, delaying oocyte maturation by 24 hours is advised.

## Abbreviations

2PN: 2 pro-nuclear; AMH: Anti-Müllerian hormone; CIR: Clinical implantation rate; CPR: Clinical pregnancy rate; GnRH: Gonadotrophin releasing hormone; GQE: Good quality embryos; hCG: Human chorionic gonadotrophin; HPE: High progesterone early group; HPL: High progesterone late group; LBR: Live birth rate; LH: Luteinizing hormone; LPE: Low progesterone early group; LPL: Low progesterone late group; MII: Metaphase 2 oocytes; OIR: Ongoing implantation rate; OPR: Ongoing pregnancy rate; PCOS: Polycystic ovary syndrome; PR: Pregnancy rate; SD: Standard deviation.

## Competing interests

The authors declare that they have no competing interests.

## Authors’ contributions

FV designed the study protocol, included patients, analysed the data and wrote the manuscript. JG included patients and helped in analysing the data. SV performed the statistical analysis. AD included patients and helped in putting the data into the database. KT helped in structuring the database. PD included patients and all authors critically revised the article and approved it to be published.
